# Supplementary material for: Heat adaptation of phage T7 under an extended genetic code
Source: Virus Evol. 2021 Dec 1;7(2):veab100. doi: 10.1093/ve/veab100 (PMC8923235; doi:10.1093/ve/veab100)
Supplement: veab100_Supp [file veab100_supp.zip › Sequencing_Analysis.zip!/Sequencing_Analysis/A1/evidence/RA_30.html]

BRESEQ :: Evidence


| Read alignment evidence... | | | | | | | | | | | |
| --- | --- | --- | --- | --- | --- | --- | --- | --- | --- | --- | --- |
|  | seq id | position | | ref | new | freq | score (cons/poly) | reads | annotation | genes | product |
| \* | NC\_001604.1 | 27,111 | 0 | T | C | 67.6% | 3289.3 / inf | 2789 | intergenic (–/–) | *–/–* | –/– |
| Reads supporting (aligned to +/- strand):  **ref** base T (358/540);  **new** base C (748/1124);  **total** (1114/1675) | | | | | | | | | | | |
| Fisher's exact test for biased strand distribution *p*-value = 9.67e-01 | | | | | | | | | | | |
| Kolmogorov-Smirnov test that lower quality scores support variant *p*-value = 7.98e-02 | | | | | | | | | | | |

Only 100 of 2834 total aligned reads displayed.  

`ACTCAGCGGTAACTTGGAGGGACGCATGGTGTACATTGGGTTCAACATTAACTTCGTATATGAGTTCTCTAAGTTCCTCATCAAGCAGACTGCCGACGACGGGTCTACCTCCACGGAAGACATTGGGCGCTTACAGTTACGCCGAGCGTGGGTTAACTACGAGAACTCTGGTACGTTTGACATTTATGTTGAGAACCAATCGTCTAACTGGAAGTACACAATGGCTGGTGCCCGATTAGGCTCTAACACTCTGAGGGCTGGGAGACTGAACTTAGGGACCGGACAATATCGATTCCCTGTGGTTGGTAACGCCAAGTTCAACACTGTATACATCTTGTCAGATGAGACTACCCCTCTGAACATCATTGGGTGTGGCTGGGAAGGTAACTACTTACGGAGAAGTTCCGGTATTTAATTAAATATTCTCCCTGTGGTGGCTCGAAATTAATACGACTCACTATAGGGAGAACAATACGACTACGGGAGGGTTTTCTTATGATGACTATAAGACCTACTAAAAGTACAGACTTTGAGGTATTCACTCCGGCTCACCATGACATTCTTGAAGCTAAGGCTGCTGGTATTGAGCCGAGTTTCCC  >  NC_001604.1/26812‑27410`  

                                                                                                                                                                                                                                                                                                           `|`                                                                                                                                                                                                                                                                                                             
  `gcAGCGGTAACTTGGAGGGACGCATGGTGTACATTGGGTTCAACATTAACTTCGTATATGAGTTCTCTAAGTTCCTCATCAAGCAGACTGCCGACGACGGGTCTACCTCCACGGAAGACATTGGGCGCTTACAGTTACGCCGAGCTTGGGTTAACTACGAGAACTCTGGTACGTTTGACATTTATGTTGAGAACCAATCGTCTAACTGGAAGTACACAATGTCTGGTGCCCGATTAGTCTCTAACACTCTGAGGGCTGGGAGACTGAACTTAGGGACCGGACAATATCGATTCCATGcggt                                                                                                                                                                                                                                                                                                          <  1:109818/300‑1 (MQ=255)`  
   `cAGCGGTAACTTGGAGGGACGCATGGTGTACATTGGGTTCAACATTAACTTCGTATATGAGTTCTCTAAGTTCCTCATCAAGCAGACTGCCGACGACGGGTCTACCTCCACGGAAGACATTGGGCGCTTACAGTTACGCCAAGCGTGGGTTAACTACGAGAACTCTGGTACGTTTGACATTTATGTTGAGAACCAATCGTCTAACT‑GAAGTACACAATGGCTGGTGCCCGATTAGGCTCTAACACTCTGAGGGCTGGGAGACTGAACTTAGGGACCGGACAATATCGATTCCCTGtggttg                                                                                                                                                                                                                                                                                                        >  1:378661/1‑301 (MQ=255)`  
     `gCGGTAACTTGGAGGGACGCATGGTGTACATTGGGTTCAACATTAACTTCGTATATGAGTTCTCTAAGTTCCTCATCAAGCAGACTGCCGACGACGGGTCTACCTTCACGGAAGACATTGGGCGCTTACAGTTACGCCGAGCGTGGGTTAACTACTAGAACTCCGGTACGTTTGACATTTATGTTGAGAACCAATCGTCTAACTGGAAGTACACAATGGCTGGTGCCCGATTAGGCTCTAACACTCTGAGGGCTGGGAGACTGAACTTAGGGACCGGACAATATCGATTCCCTGtggttgg                                                                                                                                                                                                                                                                                                       <  1:242200/301‑1 (MQ=255)`  
        `gTAACTTGGAGGGACGCATGGTGTACATTGGGTTCAACATTAACTTCGTATATGAGTTCTCTAAGTTCCTCATCAAGCAGACTGCCGACGACGGGTCTACCTTCACGGAAGACATTGGGCGCTTACAGTTACGCCGAGCGTGGGTTAACTACGAGAACTCTGGTACGTTTGACATTTATGTTGAGAACCAATCGTCTAACTGGAAGTACACAATGGCTGGTGCCCGATTAGGCTCTAACACTCTGAGGGCTGGGAGACTGAACTTAGGGACCGGACAATATCGATTCCCTGTGGTTGGTaa                                                                                                                                                                                                                                                                                                    <  1:40990/301‑1 (MQ=255)`  
          `aaCTTGGAGGGACGCATGGTGTACATTGGGTTCAACATTAACTTCGTATATGAGTTCTCTAAGTTCCTCATCAAGCAGACTGCCGACGACGGGTCTACCTCCACGGAAGACATTGGGCGCTTACAGTTACGCCGAGCGTGGGTTAACTACGAGAACTCTGGTACGTTTGACATTTATGTTGAGTACCAATCGTCTAACTGGAAGTACACAATGGCTGGTGCCCGATTAGGCTCTAACACTCTGAGGTCTGGGAGACTGAACTTAGGGACCGGTCAATATAGATTCCCTGCGGTTGTTacag                                                                                                                                                                                                                                                                                                  >  1:324131/1‑298 (MQ=255)`  
           `aCTTGGAGGGACGCATGGTGTACATTGGGTTCAACATTAACTTCGTATATGAGTTCTCTAAGTTCCTCATCAAGCAGACTGCCGACGACGGGTCTACCTCCACGGAAGACATTGGGCGCTTACAGTTACGCCGAGCGTGGGTTAACTACGAGAACTCTGGTACGTTTGACATTTATGTTGAGAACCAATCGTCTAACTGGAAGTACACAATGGCTGGTGCCCGATTAGGCCCTAACACTCTGAGGGCTGGGAGACTGAACTTAGGGACCGGACAATATCGATTCCCTGcggttctgtctct                                                                                                                                                                                                                                                                                                 >  1:438369/1‑293 (MQ=255)`  
                  `gggACGCATGGTGTACATTGGGTTCAACATTAACTTCGTATATGAGTGCTAGAAGTTCCTCATCAAGCAGACTGCCGACGACGGGTCTACCTCCACGGAAGACATTGGGCGCTTACAGTTACGCCGAGCGTGGGTTAACTACGAGAACACTGGTACGTTTGACATTTATGTTGAGAACCAATCGTCTAACTGGAAGTACACAATGGCTGGTGACCAATTAGGCTCTAACACTCTGAGGGCTGGGAGACTGAACTTAGGGACCGGACAATATCGATTCCCTGCGGTTGGTAACGCCAAGTTc                                                                                                                                                                                                                                                                                          <  1:21636/301‑1 (MQ=255)`  
                       `gCATGGTGTACATTGGGTTCAACATTAACTTCGTGTATGAGTCCTCTAAGTTCCTCATCAAGCATGCGGCCGACGACGGGTCTACCTCCACGGAAGACATTGGGCGCTTACAGTTACGCCGAGCGTGGGTTAACTACGAGAACTCTGGTACGTTTGACATTTAGGTTGAGAACCAATCGTCTAACTGGAAGTACACAATGGCTGGTGCCCGATTAGGCTCTAACACTCTGAGGGCTGGGAGACTGGACTTAGGGACCGGACAATATCGATTCCCTGCGGTTGGTAACGCCAAGTTCAacac                                                                                                                                                                                                                                                                                     <  1:194562/301‑1 (MQ=255)`  
                       `gCATGGTGTACAATGGGTTCAACATTAACTTCGTATATGAGTTCTCTAAGTTCCTCATCAAGCAGACGGCCGACGACGGGTCTACCTCCACGGAAGACATTGGGCGATTACAGTTACGCCGAGCGTGGGTTAACTACGAGAACTCTGGTACGTTTGACATATATGTTGAGAACAAATCGTCTAACTGGAAGTACACAATGGCTGGTGCCCGATTAGGCTCTAACACTCTGAGGGCTGGGAGACTGAACTTAGGGACCGGACAATATCGATTCCCTGTGGTTGGTAACGCCAAGTTCAacaa                                                                                                                                                                                                                                                                                     <  1:414552/301‑2 (MQ=255)`  
                          `gggctcggagatgtgtataagagacagtCGTATATGAGTTCTCTAAGTTCCTCATCAAGCAGACTGCCGACGACGGGTCTACCTCCACGGAAGACATTGGGCGCTTACAGTTACGCCGAGCGTGGGTTAACTACGAGAACTCTGGTACGTTTGACATTTATGTTGAGAACCAATCGTCTAACTGGAAGTACACAATGGCTGGTGCCCGATTAGGCTCTAACACTCTGAGGGCTGGGAGACTGAACTTAGGGACCTGACAATATCGATTCCCTGCGGTTGGTAACGCCAAGTTCAACACTGt                                                                                                                                                                                                                                                                                  <  1:463880/274‑1 (MQ=255)`  
                            `gtgtACATTGGGTTCAACATTAACTTCGTATATGCGTTCTCTAAGTTCCTCATCAAGCAGACTGCCGACGACGGGTCTACCTCCACTGACGACATTGGGCGCTTTCAGTTACGCCAAGCGTGGGTTAACTACGAGTACTCTGGTACGTTTGACATTTATGTTGAGAACCCATCGTCTAACTGGAAGTACACACTGGCTGTTGCCCGATTAGGCTTTAACACTCTGTGGGCTTGGTGACTGAACTTAGGTACCGGACAATATCGCTTCCCTGTGGTTGGTAACGCCAAGTTCAACACttgat                                                                                                                                                                                                                                                                                >  1:74161/1‑297 (MQ=255)`  
                            `gtgtACATTGGGTTCAACATTAACTTCGTATATGAGTTCTCTAAGTTCCTCATCAAGCAGACTGCCGACGACGGGTCTACCTCAACGGAAGACATTGGGCGCTTACAGTTACGCCGAGCGTGGGTTAACTACGAGAACTCTGGTACGTTTGACATTTATGTTGAGAACCAATCGTCTAACTGGAAGTACACAATGGCTGGTGCCCGATTAGGCTCTAACACTCTGAGGGCTGGGAGACTGAACTTAGGGACCGGACAATATCGATTCACTGTGGTTGGTAACGACACGTTCAACACTGtat                                                                                                                                                                                                                                                                                >  1:491411/1‑301 (MQ=255)`  
                            `gtgtACATTGGGTTCAACATTAACTTCGTATATGAGTGCTCTAAGTTCCTCATCAAGCAGACTGCCGACGACGGGTCTACCTTCACGGAAGACATTGGGCGCTTACAGTTACGACGAGCGTGGGTTAACTACGAGAACTCTGGTACGTTTGACATTTATGTTGAGAACCAATCGTCTAACTGGAAGTACACAATGGCTGGTGCCCGATTAGGCTCTAACACTCTGAGGGCTGGGAGACTGAACTTAGGGACCGGACAATATCGATTCCCTGTGGTTGGTAACGCCAAGTTCAACACTGtat                                                                                                                                                                                                                                                                                <  1:473156/301‑1 (MQ=255)`  
                                `tcggagatgtgtataagagacaggTATATGAGTTCTCTAAGTTCCTCATCAAGCAGACTGCCGACGACGGGTCTACCTCCACGGAAGACATTGGGCGCTTACAGTTACGCCGGGCGTGGGTTAACTACGAGAACTCTGGTACGTTTGACATTTATGTTGAGAACCAATCGTCTAACTGGAAGTACACAATGGCTGGTGCCCGATTAGGCTCTAACACTCTGAGGGCTGGGAGACTGAACTTAGGGACCGGACAATATCGATTCCCTGCGGTTGGTAACGCCAAGTTCAACACTGTATACAt                                                                                                                                                                                                                                                                            <  1:396722/278‑1 (MQ=255)`  
                                 `cATTGGGTTCAACATTAACTTCGTATATGAGTTCTCTAAGTTCCTCATCAAGCAGACTGCCGACGACGGGTCTACCTCCACGGAAGACATTGGGCGCTTACAGTTACGCCGAGCGTGGGTTAACTACGAGAACTCTGGTACGTTTGACATTTATGTTGAGAACCAATCGTCTAACTGGAAGTACACAATGGCTGGTGCCCGATTAGGCTCTAACACTCTGAGGGCTGGGAGACTGAACTTAGGGACCGGACAATATCGATTCCCTGTGGTTGGTAACGCCAAGTTCAACACTGTATACATc                                                                                                                                                                                                                                                                           <  1:388523/301‑1 (MQ=255)`  
                                 `cATTGGGTTCAACATTAACTTCGTATATGAGTTCTCTAAGTTCCTCATCAAGCAGACTGCCGACGACGGGGCTACCTCAACGGAAGACATTGGGCGCTTACAGTTACGCCGAGCGTGGGTTAACTACGAGAACTCTGGTACGTTTGACATTTATGTTGAGAACCAATCGTCTAACTGGAAGTACACAATGGCTGGTGCCCGATTAGGCTCTAACACTCTGAGGGCTGGGAGACTGAACTTAGGAACCGGACAATATCGATTCCCTGTGGTTGGTAACGCCAAGTTCAACACTGTATACATc                                                                                                                                                                                                                                                                           <  1:199355/301‑1 (MQ=255)`  
                                 `cATTGGGTTCAACATTAACTTCGTATATGAGTTCTCTAAGTGCCTCATCAAGCAGACTGCCGACGACGGGTCTACCTTCACGGAAGACATTGGGCGCTTACAGTTACGCCGAGCGTTGGTTAACTACGAGAACTCTGGTACGTTTGACATTTATGTTGAGAACCAATCGTCTAACTGGAAGTACGCAATGGCTGGTGCCCGATTAGGCTCTCACACTCTGAGGGCTGTGAGACTGAACTTAGGGACCGGACAATATCGATTCCCTGTGGTTGGTAACGCCAAGTTCAACACTGTATACATc                                                                                                                                                                                                                                                                           <  1:495115/301‑1 (MQ=255)`  
                                 `cATTGGGTTCAACATTAACTTCCTATATGAGTTCTCTAATTTCCTCCTCAAGCAGACTGCCGACGACGGGTCTTCCTCCACGGAAGACATTGTGCGCTTACAGTTACGCCGAGCGTGGGTTAACTACGAGACCTATGGTACGTTTGACATTTATGTTGATAACCAATCGTCTAACTGGAAGTACACAATGGCTGGTTCCCGATTAGGCTCTAACACTCTGAGGGCTGGGAGACTGAACTTAGGGACCGGACAATATCGATTCCCTGCGGTTGGTAACGCCAAGTTCAACACTGTATACATc                                                                                                                                                                                                                                                                           <  1:287364/301‑1 (MQ=255)`  
                                 `agacaggTTCAACATTAACTTCGTATATGAGTTCTCTAAGTTCCTCATCAAGCAGACTGCCGACGACGGGTCTACCTCCACGGAAGACATTGGGCGCTTACAGTTACGCCGAGCGTGGGTTAACTACGAGAACTCTGGTACGTTTGACATTTATGTTGAGAACCAATCGTCTAACTGGAAGTACACAATGGCTGGTGCCCGATTAGACTCTAACACTCTGAGGGCTGGGAGACTGAACTTAGGGACCGGACAATATCGATTCCCTGCGGTTGGTAACGCCAAGTTCAACACTGTATACATc                                                                                                                                                                                                                                                                           <  1:132519/296‑1 (MQ=255)`  
                                 `aaTTGGGTTCAACATTAACTTCGTATATGAGTTCTCTAAGTTCCTCATCAAACAGACTGCCGACGACGGGTCTACCTCCACGGAAGACATTGGGCGCTTACAGTTACGCCGAGCGTGTGTTAACTACGAGAACTCTGGTACGTTTGACATTTATGTTGAGAACCAATCGTCTAACTGGAAGTACACAATGGCTGGTGCCCGATTAGGCTCTAACACTCTGAGGGCTGGGAGACTGAACTTATGGACCGGACAATATCGATTCCCTGCGGTTGGTAACGCCAAGTTCAACACTGTATACATc                                                                                                                                                                                                                                                                           <  1:375042/300‑1 (MQ=255)`  
                                               `ataagagacaggatGAGTTCTCTAAGTTCCTCATCAAGCAGACTGCCGACGACGGGTCTACCTCAACGGAAGACATTGGGCGCTTACAGTTACGCCGAGCGTGGGTTAACTACGAGAACTCTGGTACGTTTGACATTTATGTTGAGAACCAATCGTCTAACTGGAAGTACACAATGGCTGGTGCCCGATTAGGCTCTAACACTCTGAGGGCTGGGAGACTGAACTTAGGGACCGGACAATATCGATTCCCTGCGGTTGGTAACGCCAAGTTCAACACTGTATACATCTTGTCAGATGAGAc                                                                                                                                                                                                                                                             <  1:229625/289‑1 (MQ=255)`  
                                                `tAACTTTGTAAATTGGTTCTCTAAGTTTCTAATCAAGGAGACTGCCGACGATGGGTTTACCTCCACGGAAGACATTGGGCGCTTACAGTTACGCAGAGCGTTGGTTAACTAAGAGAAATCTGGGAAGTTTGACATTTATGTTGAGAACCAATCGTCTAACTGGAAGTACACAATGGCTGGTGCCCGATTAGGCTCTAACACTCTGAGGGCGGGGAGACTGAACTTAGGAACCGGACAATATCGATTCCCTGCGGTTGGTAACGCCAAGTTCAACACTGTATACATCTTGTCAGATGAGACt                                                                                                                                                                                                                                                            <  1:45992/301‑1 (MQ=255)`  
                                                     `aggTATCTGAGTTCTCTAAGTTCCTCATCAAGCAGACTGCCGACGACGGGGCTACCTCCACGGAAGACATTGGGCGCTTACAGTTACGCCGAGCGTGGGTTAACTACGAGAACTCTGGTACGTTTGACATTTATGTTGAGAACCAATCGTCTAACTGGAAGTACACAATGGCTGGTGCCCGATTAGGCTCTAACACTCTGAGGGCTGGGAGACTGAACTTAGGGACCGGACAATATCGATTCCCTGCGGTTGGTAACGCCAAGTTCAACACTGTATACATCTTGTCAGATGAGACTAcccc                                                                                                                                                                                                                                                       <  1:338148/299‑1 (MQ=255)`  
                                                       `gTATATGAGTTCTCTAAGTTCCTCATCAAGCAGACTGCCGACGACGGGTCTACCTCCACGGAAGACATTGGGCGCTTACAGTTACGCCGAGCGTGGGTTAACTACGAGAACTCTGGTACGTTTGACATTTATGTTGAGAACCAATCGTCTAACTGGAAGTACACAATGGCTGGTGCCCGATTAGGCTCTAACACTCTGAGGGCTGGGAGACTGAACTTAGGGACCGGACAATATCGATTCCCTGCGGTTGGTAACGCCAAGTTCAACACTGTATACATCTTGTCAGATGAGACTACCCctc                                                                                                                                                                                                                                                     >  1:476309/1‑301 (MQ=255)`  
                                                         `gaccgAGTTCTCTAAGTTCCTCATCAATCAGACTGCCGACGACGGGTCTACCTCCACGGAAGACATTGGGCGCTTACAGTTACGCCGAGCGTTGGTTAACTACGAGCACTCTGGTACGTTTGACATTTATGTTGAGAACCAATCGTCTAACTGGAAGTACACAATGGCTGGTGCCCGATTAGGCTCTAACACTCTGAGGGCTGGGAGACTGAACTTAGGGACCGGACAATATCGATTCCCTGTGGTTGGTAACGCCAAGTTCAACACTGTATACATCTTGTCAGATGAGACTACCCCTCTg                                                                                                                                                                                                                                                   <  1:91718/297‑1 (MQ=255)`  
                                                            `tGAGTTCTCTAATTTCCTCATCAAACAGACTGCCGACGTCGGGGCTACATCCAAGGAAGACATTGGGCGTGTAACTGTACGCCGAGATTGGGTTAATTACGAGAACTTTGGTACGTTTGACATTTAGTTTGAGAACCAATCGTCTAACTGGAAGTACACAATGGCTGGTGCCCGATTAGGCTCTAACACTCTGAGGGCTGGGAGACTGAACTTAGGGACCGGACAATATCGATTCCCTGCGGTTGGTAACGCCAAGTTCAACACTGTATACATCTTGTCAGATGAGACTACCCCTCTGAAc                                                                                                                                                                                                                                                <  1:261976/301‑1 (MQ=255)`  
                                                            `gagatgtgtataagagacagtcaAGCAGACTGCCGACGACGGGTCTACCTCCACGGAAGACATTGGGCGCTTACAGTTACGCCGAGCGTGGGTTAACTACGAGAACTCTGGTACGTTTGACATTTATGTTGAGAACCAATCGTCTAACTGGAAGTACACAATGGCTGGTGCCCGATTAGGCTCTAACACTATGAGGGCTGGGAGACTGAACTTAGGGACCGGACAATATCGATTCCCTGCGGTTGGTAACGCCAAGTTCAACACTGTATACATCTTGTCAGATGAGACTACCCCTCTGAAc                                                                                                                                                                                                                                                <  1:203218/281‑1 (MQ=255)`  
                                                              `aGTTCTCTCAGGTCCTCATCAAGCAGACTGCCGACGACGGGTCTACCTCCACGGAAGACATTGGTCGCTTACATTTACGCCTAGCGTGGGTTAACTACGAGAACTCTGGTACGTTTGACATTTATGTTTAGAACCAAGCGTCTAACTGGAAGTACACAATGGCTGGTGCCCGATTAGGCTCTAACACTCTGAGGGCTGGGAGACTGAACTTAGGGACCGGACAATATCGATTCCCTGTGGTTGGTAACGCCAAGTTCAACACTGTATACATCTTGTCAGATGAGACTACCCCTCTGAAcat                                                                                                                                                                                                                                              <  1:142301/301‑1 (MQ=255)`  
                                                                 `tataagagacagtcatcaAGCAGACTGCCGACGACGGGTCTACCTCCACGGAAGACATTGGGCGCTTACAGTTACGCCGAGCGTGGGTTAACTACGAGAACTCTGGTACGTTTGACATTTATGTTGAGAACCAATCGTCTAACTGGAAGTACACAATGGCTGGTGCCCGATTAGGCTCTAACACTCTGAGGGCTGGGAGACTGAACTTAGGGACCGGACAATATCGATTCCCTGCGGTTGGTAACGCCAAGTTCAACACTGTATACATCTTGTCAGATGAGACTACCCCTCTGAAcatcat                                                                                                                                                                                                                                           <  1:30121/289‑1 (MQ=255)`  
                                                                       `tcgtgggctcggagatgtgtataagagacaGGCCTACCTCCACGGAAGCCATTGGGCGCTTACAGTTACTCCGAGCGTGGGTTAACTACGAGAACTCTGGTACGTTTGACATTTATGTTTAGAACCAATCGTCTAACTGGAAGTACACAATGGCTGGTGCCCGATTAGGCTCTAACACTCTGAGGGCTGGGAGACTGAACTTAGGGACCGGACAATATCGATTCCCTGTGGTTGGTAACGCCAAGTTCAACACTGTATACATCTTGTCAGATGAGACTACCCCTCTGAACATCATTGGgtg                                                                                                                                                                                                                                     <  1:86704/275‑1 (MQ=255)`  
                                                                           `ccTCATCAAGCAGACTGCCGACGACGGGTCTACCTCCACGGAAGACATTGGGCGCTTACAGTTACGCCGAGCGTGGGTTAACTACGAGAACTCTGGTACGTTTGACATTTATGTTGAGAACCAATCGTCTAACTGGAAGTACACAATGGCTGGTGCCCGATTAGGCTCTAACACTCTGAGGGCTGGGAGACTGAACTTAGGGACCGGACAATATCGATTCCCTGTGGTTGGTAACGCCAAGTTCAACACTGTATACATCTTGTCAGATGAGACTACCCCTCTGAACATCATTGGGTGTGGc                                                                                                                                                                                                                                 >  1:225450/1‑301 (MQ=255)`  
                                                                             `ccatcaAGCAGACTGCCGACGACGGGTCTACCTTCACGGAAGACATTGGGCGCTTACAGTTACGCCGAGCGTGGGTTAACTACGAGAACTCTGGTACGTTTGACATTTATGTTGAGAACCAATCGTCTAACGGGAAGTACACAATGGCTGGTGCCCGATTAGGCTCTAACACTCTGAGGGCTGGGAGACTGAACTTAGGGACCGGACAATATCGATTCCCTGTGGTTGGTAACGCCAAGTTCAACACTGTATACATCTTGTCAGAGGAGACTACCCCTCTGAACATCATTGGGTGTGGCTg                                                                                                                                                                                                                               <  1:83080/300‑1 (MQ=255)`  
                                                                                       `gACTTCCGACGACGGTCCTACCTCAACGGAAGACATTGGGCGCTTACAGTTACGCCGAGCGTGGGTTAACTACGAGAACTCTGGTACGTTTGACATTTATGTTGAGAACCAATCGTCTAACTGGAAGTACACAATGGCTGGTGCCCGATTAGGCTCTAACACTCTGAGGGCTGGGAGACTGAACTTAGGGACCGGACAATATCGATTCCCTGCGGTTGGTAACGCCAAGTTCAACACTGTATACATCTTGTCAGATGAGACTACCCCTCTGAACATCATTGGGTGTGGCTGGGAAGGTAac                                                                                                                                                                                                                     <  1:15114/301‑1 (MQ=255)`  
                                                                                                `agacgGGTCTACCGCCACGGAAGACATTGGGCGCTTACAGTTACGCCGAGCGTGGGTTAACTACGAGAACTCTGGTACGTTTGACATTTATGTTGAGAACCAATCGTCTAACTGGAAGTACACAATGGCTGGTGCCCGATTAGGCTCTAACACTCTGAGGGCTGGGAGACTGAACTTAGGGATCGGACAATATCGATTCCCTGTGGTTGGTAACGCCAAGTTCAACACTGTATACATCTTGTCAGATGAGACTACCCCTCTGAACATCATTGGGTGTGGCTGGGAAGGTAACTACTTACgg                                                                                                                                                                                                            <  1:146554/300‑1 (MQ=255)`  
                                                                                                 `gacgGGTCTACCTCCACGGAAGACATTGGGCGCTTACAGTTACGCCGAGCGTGGGTTAACTACGAGAACTCTGGTACGTTTGACATTTATGTTGAGAACCAATCGTCTAACTGGAAGTACACAATGGCTGGTGCCCGATTAGGCTCTAATACTCTGAGGGCTGGGAGACTGAACTTAGGGACCGGACAATATCGATTCCCTGCGGTTGGTAACGCCAAGTTCAACACTGTATACATCTTGTCAGATGAGACTACCCCTCTGAACATCATTGGGTGTGGCTGGGAAGGTAACTACTTACGga                                                                                                                                                                                                           >  1:186952/1‑301 (MQ=255)`  
                                                                                                        `cTACCTCCATGTAAGACATTGGGCGCTTACAGTTACGCCTAGCGTGGGTTAACTACGAGAACTCTGGTACGTTTGACATTTATGTTGAGAACCAATCGTCTAACTGGAAGTACACAATGGCTGGTGCCCGATTAGGCTCTAACACTCTGAGGGCTGGGAGACTGAACTTAGGGACCGGACAATATCGATTCCCTGCGGTTGGTAACGCCAAGTTCAACACTGTATACATCTTGTCAGATGAGACTACCCCTCTGAACATCATTGGGTGTGGCTGGGAAGGTAACTACTTACGGAGAAGTTc                                                                                                                                                                                                    <  1:365007/301‑1 (MQ=255)`  
                                                                                                             `cccACTGAAGAAATTGTGAGCTTACAGTTACGCCGAGCGTGGGTTAACTACGAGAACTCTGGTACGTTTGACATTTATGTTGAGAATCAATCGTCTAACTGGAAGTACACAATGGCTGGTGCCCGATTAGGCTCTAACACTCTGAGGGCTGGGAGACTGAACTTAGGGACCGGACAATATCGATTCCCTGCGGTTGGTAACGCAAAGTTCAACACTGTATACATCTTGTCAGATGAGACTACCCCTCTGAACATCATTGGGTGTGGCTGGGAAGGTAACTACTTACGGAGAAGTTCCGGTa                                                                                                                                                                                               <  1:274365/300‑1 (MQ=255)`  
                                                                                                                `aCGGAAGACATTGGGCGCTTACAGTTACGCCGAGCGTGGGTTAACTCCGAGAACTCTGGTACGTTTGACATTTATGATGAGAACCAATCGTCTAACTGGAAGTACACAATGGATAGTGCCCGATTAGGCACTAACACCCTGAGGGCTGGGAGACTGAACTTAGGGACCGGAACATATCGATTCCATGCGGTTGGTAACGCAAAGTTCAACACTGTATACATATTGTCAGATGAGACTACCCCTCTGAACATCATTGGGTGTGGCTGGGAAGGGAACGACTTACGgagcggactctcataca                                                                                                                                                                                            >  1:314035/1‑287 (MQ=255)`  
                                                                                                                   `gAAGACATTGGGCGCTTACAGTTACGCCGAGCGTGGGTTAACTACGAGAACTCTGGTACGTTTGACATTTATGTTGCGAAACAATCGTCTAACTGGAAGTACACAATGGCTGGTGCCCGATTAGGCTCTAACACTCTGAGGGCTGGGAGAATGAACTTAGGGACCGGACAATATCGATTTCCTGCGGTTGGTAAAGCCAAGTTAAACACTGTAGACATCTTGACAGATGAGACTAACCCTATGAAAACAAATGGATGTAGCTGGGAAGGATACTACTTAAGGAGAAGtctgtctcatctac                                                                                                                                                                                         >  1:284231/1‑288 (MQ=255)`  
                                                                                                                   `gAAGACATTGGGCGCTTACAGTTACGCCGAGCGTGGGTTAACTACGAGAACTCTGGTACGTTTGACATTTATGTTGAGAACCAATCGTCTAACTGGAAGTACACAATGGCTGGTGCCTGATTAGGCTCTAACACTCTGAGGGCTGGGAGACTGAACTTAGGGACCGGACAATATCGATTCCCTGCGGTTGGTAACGCCAAGTTCAACACTGTATACATCTTGTCAGATGAGACTACCCCTCTGAACATCATTGGGTGTGGCTGGGAAGGTAACTACTTACGGAGAAGTTCCTGTATttaat                                                                                                                                                                                         >  1:26729/1‑301 (MQ=255)`  
                                                                                                                        `cATTGGGCGCTTACAGTTACGCCGAGCGTGGGTTAACTACGAGAACTCTGGTACGTTTGACATTTATGTTGAGAACCAATCGTCTAACTGGAAGTACACAATGGCTGGTGCCCGATTAGGCTCTAACACTCTGAGGGCTGGGAGACTGAACTTAGGGAACGGACAATATCGATTCCCTGTGGTTGGTAACGCCAAGTTCAACAATGTATACATATTGTCAGATGAGACTACCCCTCTGAAAATCATTGGGTGTGGATGGGAAGGTAACTAATTACGGAAAAGGTTCGGTATTTAATTAAat                                                                                                                                                                                    >  1:127668/1‑301 (MQ=255)`  
                                                                                                                                `gacagcAGTTACGCCGAGCGTGGGTTAACTACGAGAACTCTGGTACGTTTGACATTTATGTTGAGAACCAATCGTCTAACTGGAAGTACACAATGGCTGGTGCCCGATTAGGCTCTAACACTCTGAGGGCTGGGAGACTGAACTTAGGGACCGGACAATATCGATTCCCTGCGGTTGGTAACGCCAAGTTCAACACTGTATACATCTTGTCAGATGAGACTACCCCTCTGAACATCATTGGGTGTGTCTGGGAAGGTAACTACTTACGGAGAAGTTCCTGTATTTAATTAAATATTCTccc                                                                                                                                                                            <  1:447867/296‑1 (MQ=255)`  
                                                                                                                                     `cAGTTACGCCGAGCGTGGGTTAACTACGAGAACTCTGGTACGTTTGACATTTATGTTGAGAACCAATCGTCTACCTGGAAGTACAGAATGGCTGGTTCCCGATTAGGCTCTAACACTCTGAGGTCTTGGCGACTGAACTTAGGGACCGTACACTTTCGATTCCTTTCTGTTGGTAACGCCAAGTTCAACACTGTATACATCTTCTCTAATTATACTACCCCTCTTAACATCACTGGATGTGGCTGTTTATTTACCTAATTACGGAGAGGTTACGATATTTAATTCACTATTCTACCttttt                                                                                                                                                                       >  1:252126/1‑297 (MQ=255)`  
                                                                                                                                      `aGTTACGCCGAGCGTGGGTTAACTACGAGAACTCGGGTACGTTTGACATTTATGTTGAGAACCAATCGTCTAACTGGAAGTACACAATGGCTGGTGCCCGATTAGGCTCTAACACTCTGAGGGCTGGGAGACTGAACTTAGGGACCGGACAATATCGATTCCCTGCGGTTGGTAACGCCAAGTTCAACACTGTATACATCTTGTCAGATGAGACTACCCCTCTGAACATCATTGGGTGTGGCTGGGAAGGTAACTACTTACGGAGAAGTTCCGGTATTTAATTAAATATTCTACCTgtggt                                                                                                                                                                      <  1:486337/301‑1 (MQ=255)`  
                                                                                                                                       `gTTACGCCGAGCGTGGGTTAACTACGAGAACTCTGGTACGTTTGACATTTATGTTCAGAACCAATCGTCTAACTGGAAGTACACAATGGCTGGTGCCCGATTAGGCTCTAACACTCTGAGGGCTGGGAGACTGAACTTAGGGACCGGACAATATCGATTCCCTGCGGTTGGTAACGCCACGTTCAACACTGTATACATCTTGTCAGATGAGACTACCCCTCTGAACATCATTGGGTGTGGCTGGGAAGGTAACTACTTACGGAGCAGTTCCGGTATTTAATTAAATATTCTCCCTgtggtg                                                                                                                                                                     >  1:166362/1‑301 (MQ=255)`  
                                                                                                                                               `gAGCGTTGGTTAACTACGAGAACTCTGGTACGTTTGACATTTATGTTGAGAACCAATCGTCTAACTGGCAGTACACAATGGCTGGTGCCCGATTAGGCTCTAACACTCTGAGTGCTGGGAGACTGAACTTAGGGACCGGACAATATCGATTCCCTGCGGTTGGTAACGCCCAGTTCAACACTGTATACATCTTGTCAGATGAGACTACCCCTCTGAACATCCTTGGGTGTGGCTTGTACGGTAACTACTTACGGAGAAGTTCCGGTATTTCATTAACTTTTCTCCCTGTCGTGGCTCGaaa                                                                                                                                                             >  1:468746/1‑301 (MQ=255)`  
                                                                                                                                               `gAGCGTGGGTTAACTACTAGAACTCTGGTACGTTTGACATTTATGTTGAGAACCAATCGACTAACTGGAAGTACACAATGGCTGGTGCCCGATTAGGCTCTAACACTCTGAGGGCTGGGAGACTGACCTTAGGGACAGGACAATATCGATTCCCTGCGGTTGGTAACGCCAAGTTCAACACTGTATACATCTGGTCAGATGAGACTACCCCTCTGAACATAATTGGGTGTGGCTGGGAAGGTAACTAATTACGGAGAAGTTCCGGTATTTAATTAAATATTCTCACTGTGGTGGCTCTaaa                                                                                                                                                             >  1:361292/1‑301 (MQ=255)`  
                                                                                                                                                 `gCGTGGGTTAACTACGAGAACTCTGGTACGTTTGACATTTATGTTGAGAACCAATCGTCTAACTGGAAGTACACAATGGCTGGTGCCCGATTAGGCTCTAACACTCTGAGGGCTGGGAGACTGAACTTAGGCACCGGACAATATCGATTCCATGCGGTTGGTAACGCCCAGTTCAACACTGTATACCTCTTGTCAGATGAGACTACCCCTCTGAACATCATTTTGTTTGGCTGGGAAGGTAACTACTTACGGATATGTTCCGTTATTTAATTAAATATTCTCCCTCTTGTTGCTCGAAAtt                                                                                                                                                           >  1:401612/1‑301 (MQ=255)`  
                                                                                                                                                    `tGGGTTAACTACGAGAACTCTGGTACGTTTGACATTTATGTTGAGAACCAATCGTCTAACTGGAAGTACACAATGGCTGGTGCCCGATTAGGCTCTAACACTCTGAGGGCTGGGAGACTGAACTTAGGGACCGGACAATATCGATTCCCTGCGGTTGGTAACGCCAAGTTCAACACTGTATACATCTTGTCAGATGAGACTACCCCTCTGAACATCATTGTGTGTGGCTGGGAAGGTAACTACTTACGGAGAAGTTACGGTATTTAATTAAATATTCTCCCTGTGGTGGATCGAAATTAAt                                                                                                                                                        >  1:66093/1‑301 (MQ=255)`  
                                                                                                                                                      `ggTTAACTACGAGAACTCTGGTACGTTTGACATTTATGTTGAGAACCAATCGTCTAACTGGAAGTACACAATGGCTGGTGCCCGATTAGGCTCTAACACTCTGAGGGCTGGGAGACTGAACTTAGGGACCGGACAATATCGATTCCCTGTGGTTGGTAACGCCAAGTTCAACACTGTATACATCTTGTCAGATGAGACTACCCCTCTGAACATCATTGGGTGTGGCTGGGAAGGTAACTACTTACGGAGAAGTTCCGGTATTGAATTAAATATTCTCCCTGTGGTGGCTCGCAGTtactac                                                                                                                                                      >  1:481564/1‑301 (MQ=255)`  
                                                                                                                                                      `ggTTAACTACGAGAACTCTGGTACGTTTGACATTTATGTTGAGAACCAATCGTCTAACTGGAAGTACAAAATGGCTGGTGCCCGATTAGGCTCTAACACTCTGAGGGCTGGGAGACTGAACTTAGGGACCGTACAATATCGATTCCCTGCGGTTGGTAACGCCAAGTTCAACACTGTATACATCTTGTCAGATGAGACTACCCCTCTGAACATCATTGGGTGTGGCTGGGAAGGTAACTACTTACGGAGAAGTTCCGGTATTTAATTAAATATTCTCCCTGTGGTGGCTCGAAATTAATAc                                                                                                                                                      <  1:10550/301‑1 (MQ=255)`  
                                                                                                                                                      `agagacagaCGAGAACTCTGGTACGGTGGACATTTATGTTGAGAACAAATCGTCTAACTGGAAGTACACAATGGCTGGTGCCCGATTAGGCTCTAACACTCTGAGGGATGGGAGACTGAACTTAGGGACCGGACAATATCGATTCCCTGCGGTTGGTAACGCCAAGTTCAACACTGTATACATCTTGTCAGATGAGACTACCCCTCTGAACATCATTGGGTGTGGCTGGGAAGGTAACTACTTACGGAGAAGTTCCGGTATTTAATTAAATATTCTCCCTGTGGTGGCTCGAAATTAATAc                                                                                                                                                      <  1:453154/293‑1 (MQ=255)`  
                                                                                                                                                            `cTACGAGAACTCTGGTACGTTTTACATTTATTTTGAGAACCAATCGTCTAACTGGAAGTACACAATGGCTGGTGCCCGATTAGGCTCTAACACTCTGAGGGCTGGGAGACTGAACTTAGGGACCGGACAATATCGATCCCCTGCGGTTGGTAAAGCCAAGTTCAACACTATATACATCTTGTCTGATGAGACTACCCCTCTGAACATCATTGGTTGTCGCTGGGAAGGTAACTACTTACGGAGAAGTTCCGGTATTTAATTAAATATTCTCACTGTGGTGGCTCGAAATTAATACGACTcc                                                                                                                                                >  1:211433/1‑300 (MQ=255)`  
                                                                                                                                                                `gacagctctGGTACGTTTGACATTTATGTTGAGAACCAATCGTCTAACTGGAAGTACACAATGGCTGGTGCCCGATTAGGCTCTAACACTCTGAGGGCTGGGAGACTGAACTTAGGGACCGGACAATATCGATTCCCTGCGGTTGGTAACGCCAAGTTCAACACTGTATACATCTTGTCAGATGAGACTACACCTCTGAACATCATTGGGTGTGGCTGGGAAGGTAACTACTTACGGAGAAGTTCCGGTATTTAATTAAATATTCTCCCTGTGGTGGCTCGAAATTAATACGACTCACtat                                                                                                                                            <  1:220556/296‑1 (MQ=255)`  
                                                                                                                                                                `gaaaaCTCTGATACGTTTGACATTTATGTTGAGTACCAATCTTCTAACTGGAAGTACACAATGGATGGTGCCCGATTAGGCTCGAACACTCTGATAGCTTGGAGACTGAACTTAGGGACCGGACAATATCGATTCCCTGTGGTTGGTAACGCCAAGTTCAACACTGTATACATCTTGTCAGATGAGACTACCCCTCTGAACATCATTGGGTGTGGATGGGAAGGTAACTACTTACGGAGAAGTTCCGGTATTTAATTAAATATTCTCCCTGTGGTGGCTCGAAATTAATACGACTAACtat                                                                                                                                            <  1:95766/298‑1 (MQ=255)`  
                                                                                                                                                                    `tgggctcggagatgtgtataagagacaggaACCAATCGTCTAACTGGAAGTACACAATGGCTGGTGCCCGATTAGGCTCTAACACTCTGAGGGCTGGGAGACTGAACTTAGGGACCGGACAATATCGATTCCCTGTGGTTGGTAACGCCAAGTTCAACACTGTATACATCTTGTCAGATGAGACTACCCCTCTGAACATCATTGGGTGTGGCTGGGAAGGTAACTACTTACGGAGAAGTTCCGGTATTTAATTAAATATTCTCCCTGTGGTGGCTCGAAATTAATACGACTCACTATAggg                                                                                                                                        <  1:13699/273‑1 (MQ=255)`  
                                                                                                                                                                    `gtgtataagagacagACATTTATGTTGAGAAACAATCGTCTAACTGGAAGTACACAATGGCTGGTGCCCGATTAGGCTCTAACACTCTGAGGGCTGGGAGACTGAACTTAGGGACCGGACAATATCGATTCCCTTCGGTTGGTAACGCCAAGTTCAACACTGTATACATCTTGTCAGATGAGACTACCCCTCTGAACATCATTGGGTGTGGCTGGGAAGGTAACTACTTACGGAGAAGTTCCGGTATTTAATTAAATATTCTCCCTGTGGTGGCTCGAAATTAATACGACTCACTATAggg                                                                                                                                        <  1:382911/287‑1 (MQ=255)`  
                                                                                                                                                                    `aCTCTGGTACGTTTGACATTTATGTTGAGAACCAATCGTCTAACTGGAAGTACACAATGGCTGGTTCCCGATTAGGCTCTAACACTCTGAGGGCTGGGAGACTGAACTTAGGGACCGTACAATATCGATTCCCTGCGGTTGGTAACGCCCAGTTCAACACTCTATACATCTTGTCAGATGAGACTCCCCCTCTGAACATCATTTGGTGTGGCTGGGAAGGTAACTACTTACGGAGAAGTTCCGGTATTTAATTAAATATTCTCCCTGTGGTTGCTCGAAATTAATACGACTCACTCTTggg                                                                                                                                        >  1:57835/1‑301 (MQ=255)`  
                                                                                                                                                                    `aCTCTGGTACGTTTGACATTTATGTTGAGAACCAATCGTCTAACTGGAAGTACACAATGGCTGGTGCCCGATTAGGCTCTAACACTCTGAGGGCTGGGAGACTGAACTTAGGGACCGGACAATATCGATTCCCTGCGGTTGGTAACGCCAAGTTCAACACTGTATACATCTTGTCAGATGAGACTACCCCTCTGAACATCATTGGGTGTGGCTGGGAAGGTAACTACTTACGGAGAAGTTCCGGTATTTAATTAAATATTCTCCCTGTGGTGGCTCGAAATTAATACGACTCACTATAggg                                                                                                                                        <  1:444557/301‑1 (MQ=255)`  
                                                                                                                                                                    `aCTCTGGTACGTTTGACATTTATGTGGAGAACCAATCGTCTAACTGGAAGTACACAATGGCTGGTGCCCGATTAGGCTCTAACACTCTGAGGGCTGGGAGACTGAACTTAGGGACCGGACAATATCGATTCCCTGCGGTTGGTAACGCCAAGTTCAACACTGTATACATCTTGTCAGATGAGACTACCCCTCTGAACATCATTGGGTGTGGCTGGGAAGGTAACTACTTACGGAGAAGTTCCGGTATTTAATTAAATATTCTCCCTGTGGTGGCTCGAAATTAATACGACTCACTATAggg                                                                                                                                        <  1:238946/301‑1 (MQ=255)`  
                                                                                                                                                                    `aCTCTGGTACGTTTGACATTTATGGTGAGAACCAATCGTCTAACTGGAAGTACACAATGGCTGGTGCCCCATTAGGCTCTAACACTCTGAGGGCTGGGAGACTGAACTTAGGGACCGGACAATATCGATTCCCTGCGGTTGGTAACGCCAAGTTCAACACTGTATACATCTTGTCAGATGAGACTACCCCTCTGAACATCATTGGGTGTGGCTGGGAAGGTAACTACTTACGGAGAAGTTCCGGTATTTAATTAAATATTCTCCCTGTGGTGGCTCGAAATTAATACGACTCACTATAggg                                                                                                                                        <  1:333795/301‑1 (MQ=255)`  
                                                                                                                                                                    `aCTCTGGTACGGTTGAAATGTATGTTAAGAACCAATCGTCTAACTGGAAGTACACAATGGCTGGTGACCCATTAGTCTCTAACAATCTGGGGGCTGGAAGACTGAACTTATGGACCGGACAATATCGATTCCCTTCGGTTGGTAACTCCAAGTTCAACACTGTATACATCTTGTCAAATGAGACTACCCCTCTGAACATCATTGGGTGTGGCTGGGAAGGTAACTACTTACGGAGAAGTTCCGGTATTTAATTAAATATTCTCCCTGTGGTGGCTCGAAATTAATACGACTCACTATAggg                                                                                                                                        <  1:292809/301‑1 (MQ=255)`  
                                                                                                                                                                     `ctctGGTACGTTTGACATTTATGTTGAGAACCAATCGTCTAACTGGAAGTACACAATGGCTGGTGCCCGATTAGGCTCTAACACTCTGAGTGCTGGGAGACTGAACTTAGGGACCGGACAATATCGATTCCCTGCGGTTGGTAACGCCAAGTTCAACACTGTATAAATCTTGTCAGTTTAGACTACCCCTCTGAACATCATTGGGTGTGGCTGGGAAGGTAACTACTTACGCATAAGTTCCGGTATTTAATTAATTATTGTCCCTTTTGTGTCTCGAAATTAATACTAATAACTATAggtg                                                                                                                                       >  1:352000/1‑299 (MQ=255)`  
                                                                                                                                                                     `ctctGGTACGTTTGACATTTATGTTGAGAACCAATCGTCTAACTGGAAGTACACAATGGCTGGTGCCCGATTAGGCTCTAACACTCTGAGGGCTGGGAGACTGAACTTAGTGACCGGACAATATCGATTCCCTGCGGTTGGTAACGCCAAGTTCAACACTGTATACATCTTGTCAGATGAGACTACCCCTCTGAACATCATTGGGTGTGGCTGGGAAGGTAACTACTTACGGAGAAGTTCCGGTATTTAATTAAATATTCTCCCTGTGGTGGCTCGAAATTAATACGAcctgtctcttata                                                                                                                                       >  1:12255/1‑289 (MQ=255)`  
                                                                                                                                                                     `ctctGGTACGTTTGACATTTATGTTGAGAACCAATCGTCTAACTGGAAGTACACAATGGCTGGTGCCCGATTAGGCTCTAACACTCTGAGGGCTGGGAGACTGAACTTAGGGACCGGACAATATCGATTCCCTGCGGTTGGTAACGCCAAGTTCAACACTGTATACATCTTGTCAGATGAGACTACCCCTCTGAACATCATTGGGTGTGGCTGGGAAGGTAACTACTTACGGAGAAGTTCCGGTATTTAATTAAATATTCTCCCTGTGGTGGCTCGAAATTAATACGACTCACTATAGGga                                                                                                                                       <  1:355994/301‑1 (MQ=255)`  
                                                                                                                                                                         `ggTACGTTTGACATTTATGTTGAGAACCAATCGTCTAACTGGAAGTACACAATGGCTGGTGCCCGATTAGGCTCTAACACTCTGAGGGCTGGGAGACTGAACTTAGGGACCGGACAATATCGATTCCCTGTGGTTGGTAACGCCATGTTCAACACTGTATACATCTTGTCAGATGAGACTACCCCTCTGAACATCATTGGGGTTGGCTGGGAAGGTAAATACTTACGGAGAAGTTCCGGTATTTCATTAAATATTCTCCCTGTGTTGGCTCGAAATTAATACGACTCACtatatgtataaa                                                                                                                                   >  1:15566/1‑293 (MQ=255)`  
                                                                                                                                                                             `cGTTTGACATTTATGTTGAGAACCAATCGTCTAACTGGAAGTAAACAATGGCTGGTGCCCGATTAGGCTCTAACACTCTGCGGGCGGGGAGACTGAACTTAGGGACCGGACAATACCGCTTCCCTGAGGTTGGTAACGCCAAGTTCAACACTGTATACATCTTGTCAGATGAGCTTACTCCTCTGAACCTCATTGGGTGTGGCGTGGACGGTAATTACTTACGTAGAAGTTCCGGTATTTAATTAAATATTCTCCCTGTGTTGGCTCGTAATTCATACGatcccccataggacttgctctt                                                                                                                               >  1:329081/1‑280 (MQ=255)`  
                                                                                                                                                                               `tttGACATTTATGTTGAGAACCAATCGTCTAACTGGAAGTACACAATGGCTGGTGCCCGATTAGGCTCTAACACTCTGAGGGCTGGGAGACTGAACTTAGGGACCGGACAATATCGATTCCCTGCGGTTGGTAACGCCAAGTTCAACACTGTATACATCTTGTCAGATGAGACTACCCCTCTGAACATCATTGGGTGTGGCTGGGAAGGTAACTACTTACGGAGAAGTTCCGGTATTTAATTAAATATTCTCCCTGTGGTGGCTCGAAATTAATACGACTCACTATAGGGAGAACAATACg                                                                                                                             >  1:114600/1‑301 (MQ=255)`  
                                                                                                                                                                                    `cATTTATGTTGAGAACCAATCGTCTAACTGGAAGTACACAATGGCTGGTGCCCGATTAGGCTCTAACACTCTGAGGGCTGGGAGACTGAACTTAGGGACCGGACAATATCGATTCCCTGCGGTTGGTAACGCCAAGTTCAACACTGTATACATCTTGTCAGATGAGACTACCCCTCTGAACATCATTGGGTGTGGCTGGGAAGGTAACTACTTACGGAGAAGTTCCGGTATTTAATTAAATATTCTCCCTGTGGTGGCTCGAAATTAATACGACTCACTATAGGGAGAACAATACGACTaa                                                                                                                        >  1:371545/1‑300 (MQ=255)`  
                                                                                                                                                                                      `tttATGTTGAGAACCAATCGTCTAACTGGAAGTACACAATGGCTGGTGCCCGATTAGGCTCTAACACTCTGAGGGCTGGGAGACTGAACTTAGGGACCGGACAATATCGATTCCCTGCGGTTGGTAACGCCAAGTTCAACACTGTATCCATCTTGTCAGATGAGACTACCCCTCTGAACATCATTGGGTGTGGCTGGGAAGGTAACTACTTACGGAGAAGTTCCGGTATTTAATTAAATATTCTCCCTCTGGTGGCTCGAAATTAATACGACTAACTATAGGGTGAACAATACGagtcagg                                                                                                                      >  1:257468/1‑295 (MQ=255)`  
                                                                                                                                                                                         `aTGTTGAGAACCAATCGTCTAACTGGAAGTACACAATGGCTGGTGCCCGATTAGGCTCTAACACTCTGAGGGCTGGGAGACTGAACTTAGGGACCGGACAATATCGATTCCCTGCGGTTGGTAACGCCAAGTTCAACACTGTATACATCTTGTCAGATGAGACTACCCCTCTGAACATCATTGGGTGTGGCTGGGAAGGTAACTACTTACGGAGAAGTTCCGGTATTTAATTAAATATTCTCCCTGTGGTGGCTCGAAATTAATACGACTCACTATAGGGAGAACAATACGACTACGGGAg                                                                                                                   >  1:160819/1‑301 (MQ=255)`  
                                                                                                                                                                                           `gTTGAGAACCAATCGTCTAACTGGAAGTACACAATGGCTGGTGCCCGATTAGGCTCTAACACTCTGAGGGCTGGGAGAATGAACTTAGGGACCGGACAATATCGATTCCCTGCGGTTGGTAACGCCATGTTCAACACTGTATACATCTTGTCAGATGAGACTACCCCTCTGAACATCATTGGGTGTGGCTGGGAAGGTAACTACTTACGGAGAAGTTCCGTTATTTAATTAAATATTCTCCCTGTGGTGGCTCGAAATTCATACGACTCACTATAGGGAGAACAATACTACTACGGGAggg                                                                                                                 >  1:347130/1‑301 (MQ=255)`  
                                                                                                                                                                                                   `ccAATCGTCTAACTGGAAGTACACAATGGCTGGTGCCCGATTAGGCTCTAACACTCTGAGGGCTGGGAGACTGAACTTAGGGACCGGACAATATCGATTCCCTGTGGTTGGTAACGCCAAGTTCAACACTGTATACATCTTGTCAGATGAGACTACCCCTCTGAACATCATTGGGTGTGGCTGGGAAGGTAACTACTTACGGAGAAGTTCCGGTATTTAATTAAATATTCTCCCTGTGGTGGCTCGAAATTAATACGACTCACTATAGGGAGAACAATAAGACTACGGGAGGGTTTTCTTa                                                                                                         >  1:177280/1‑301 (MQ=255)`  
                                                                                                                                                                                                   `acAATCGTCTAACTGGAAGTACACAATGGTTGGTGCACGATTAGGCTCTAACACTCTGAGGGCTGGGATACTGAACTTAGGGACCGGAAAATATCGATTCCCTGCGGTTGGTAACGCCAAGTTCAACACTGTATACATCTTGTCAGATGAGACTACCCCTCTGAACATCATTGGGTGTGGCTGGGAAGGTAACTACTTACGGAGAAGTTCCGGTATTTAATTAAATATTCTCCCTGTGGTGGCTCGAAATTAATACGACTCACTATAGGGAGAACAATACGACTACGGGAGGGTTTTCTTa                                                                                                         <  1:100070/300‑1 (MQ=255)`  
                                                                                                                                                                                                          `tCTAACTGGAAGTACACAATGGCTGGTGCCCGATTAGGCTCTAACACTCTGAGGGCTGGGAGACTGAACTTAGGGACCGGACAATATCGATTCCCTGCGGTTGGTAACGCCAAGTTCAACACTGTATACATCTTGTCAGATGAGACTACCCCTCTGAACATCATTGGGTGTGGCTGGGAAGGTAACTACTTACGGAGAAGTTCCGGTATTTAATTAAATATTCTCCCTGTGGTGGCTCGAAATTAATACGACTCACTATAGGGAGAACAATACGACTACGGGAGGGTTTTCTTATGATGAc                                                                                                  <  1:172322/301‑1 (MQ=255)`  
                                                                                                                                                                                                             `aaCTGGAAGTACACAATGGCTGGTGCCCGATTAGGCTCTAACACTCTGAGGGCTGGGAGACTGAACTTAGGGACCGGACAATATCGATTCCCTGCGGTTGGTAACGCCAAGTTCAACACTGTATACATCTTGTCAGATGAGACTACCCCTCTGAACATCATTGGGTGTGGCTGGGAAGGTAACTACTTACGGAGAAGTTCCGGTATTTAATTAAATATTCTCCCTGTGGTGGCTCGAAATTAATACGACTCACTATAGGGAGAACAATACGACTACGGGAGTGTTTTCTTATGATGACtat                                                                                               >  1:206603/1‑301 (MQ=255)`  
                                                                                                                                                                                                                  `gAAGTACACAATGGCTGGTGCCCGATTAGGCTCTAACACTCTGAGGGCTGGGAGACTGAACTTAGTGACCGGACAATATCGATTCCCTGTGGTTGGTAACGCCAAGTTCAACACTGTATACATCTTGTCAGATGAGACTACCCCCCTGAACATCATTGGGTGTGTCTGGGAAGGTAACTACTTACGGAGAAGTTCCGGTATTTAATTAAATATTCTCCCTGTGGTGGCTCGAAATTAATACGACTCACTATAGGGAGAACAATACGACTACGGGAGGGTTTTCTTATGATGACTATAAGAc                                                                                          <  1:422724/301‑1 (MQ=255)`  
                                                                                                                                                                                                                  `gAAGTACACAATGGCTGGTGCCCGATTAGGCTCTAACACTCTGAGGGCTGGGAGACTGAACTTAGGGACCGGACAATATCGATTCCCTGCGGTTGGTAACGCCAAGTTCAACACTGTATACATCTTGTCAGATGAGACTACCCCTCTTAACATCATTGGGTGTGGCTGGGAAGGTAACTACTTACGGAGAAGTTCCGGTATTTAATTAAATATTCTCCCTGTGGTGGCTCGAAATTAATACGACTCACTATAGGGAGAACAATACGACTACGGGAGGGTTTTCTTATGATGACTATAAGAc                                                                                          >  1:435029/1‑301 (MQ=255)`  
                                                                                                                                                                                                                  `atgtgtataagagacaggggCCCGATTAGGCTCTAACACTCTGAGGGCTGGGAGACTGAACTTAGGGACCGGACAATATCGATTCCCTGCGGTTGGTAACGCCAAGTTCAACACTGTATACATCTTGTCAGATGAGACTACCACTCTGAACATCATTGGGTGTGGCTGGGAAGGTAACTACTTACGGAGAAGTTCCGGTATTTAATTAAATATTCTCCATGTGGTGGCTCGAAATTAATACGACTCACTATAGGGAGAACAATACGACTACGGGAGGGTTTTCTTATGATGACTATAAGAc                                                                                          <  1:1910/282‑1 (MQ=255)`  
                                                                                                                                                                                                                  `agagaaagcaATGGCTGGTGCCAGATTAGGCTCTAACACTCTGAGGGCTGGGAGACTGAACTTAGGGACCGGACAATATCGATTCCCTGCGGTTGGTAACGCCAAGTTCAACACTGTATACATCTTGTCAGATGAGACTACCCCTCTGAACATCATTGGGTGTGGCTGGGAAGGTAACTACTTACGGAGAAGTTCCGGTATTTAATTAAATATTCTCCCTGTGGTGGCTCGAAATTAATACGACTCACTATAGGGAGAACAATACGACTACGGGAGGGTTTTCTTATGATGACTATAAGAc                                                                                          <  1:500883/293‑1 (MQ=255)`  
                                                                                                                                                                                                                     `gTACACAATGGCTGGTGCCCGATTAGGCTCTAACACTCTGAGGGCTGGGAGACTGAACTTAGGGACCTGACAATATCGATTCCCTGCTGTTGGTAACGCCAAGTTCAACACTGTATACATCTTGTCAGATGAGACTACCCCTCTGAACATCATTGGGTGTGGCTGGGAAGGTAACTACTTACGGAGAAGTTCCGGTATTTAATTAAATATTCTCCCTGTGGTGGCTCGAAATTAATACGACTCACTATAGGGAGAACAATACGACTACGGGAGGGTTTTCTTATGATGACTATAAGACcta                                                                                       >  1:431195/1‑301 (MQ=255)`  
                                                                                                                                                                                                                     `gTACACAATGGCTGGTGCCCGATTAGGCTCTAACACTCTGAGGGCTGGGAGACTGAACTTAGGGACCGGACAATATCGATTCCCTGCGGTTGGTAACGCCAAGTTCAACACTGTATACATCTTGTCAGATGAGACTACACCTCTGAACATCATTGGGTGTGGCTGGGAAGGTAACTACTTACGGAGAAGTTCCGGTATTTAATTAAATATTCTCCCTGTGGTGGCTCGAAATTAATACGACTCACTATAGGGAGAACAATACGAATACGGGAGGGTTTTCTTATGATGACTATAAGACctt                                                                                       >  1:61200/1‑300 (MQ=255)`  
                                                                                                                                                                                                                        `cacaATGGCTGGTGCCCGATTAGGCTCTAACACTCTGAGGGCTGGGAGACTGAACTTAGGGACCGGACAATATCGATTCCCTGCGGTTGGTAACGCCAAGTTCAACACTGTATACATCTTGTCAGATGAGACTACCCCTCTGAACATCATTGGGTGTGGCTGGGAAGGTAACTACTTACGGAGAAGTTCCGGTATTTAATTAAATATTCTCTCTGTGGTTGCTCGAAATTAATACGACTCACTATAGGGAGAACACTACGACTACGGGAGGGTTTGCTTATGATTACTATAAGAActacta                                                                                    >  1:278526/1‑301 (MQ=255)`  
                                                                                                                                                                                                                          `aaaTGGCTGGTGACCGATTAGGCTCTAACACTCTGAGGGCTGGGAGACTGAACTTAGGGACCGGACAATATCGATTCCCTGCGGTTGGTAACGCCAAGTTCAACACTGTATACATCTTGTCAGATGAGACTACCCCTCTGAACATCATTGGGTGTGGCTGGGAAGGTAACTACTTACGGAGAAGTTCCGGTATTTAATTAAATATTCTCCCTGTGGTGGCTCGAAATTAATACGACTCACTATAGGGAGAACAATACGACTACGGGAGGGTTTTCTTATGATGACTATAAGACCTACTaaa                                                                                  <  1:119355/300‑1 (MQ=255)`  
                                                                                                                                                                                                                                        `cGATTAGGCTCTAACACTCTGAGGGCTGGGAGACTGAACTTAGGGACCGGACAATATCGATTCCCTGCGGTTGGTAACGCCAAGTTCAACACTGTATACATCTTGTCAGATGAGACTACCCCTCTGAACATCATTGGGTGTGGCTGGGAAGGTAACTACTTATGGAGAAGTTCCGGTATTTAATTAAATATTCTCCCTGTGGTGGCTCGAAATTAATACGACTCACTATAGGGAGAACAATACGACTACGGGAGGGTTTTCTTATGATGACTATAAGACCTACTAAAAGTACAGACTTTGa                                                                    >  1:342566/1‑301 (MQ=255)`  
                                                                                                                                                                                                                                          `aTTAGGCTCTAACACTCTGAGGGCTGGTAGACTGAACTTAGGGACCGGACAATATCGATTCCCTGCGGTTGGTAACGCCAAGTTCAACACTGTATACATCTTGTCAGATGAGACTACCCCTCTGAACATCATTGGGTGTGGCTGGGAAGGTAACTACTTACGGAGAAGTTCCGGTATTTAATTAAATATTCTCCCTGTGGTGGCTCGAAATTAATACGACTCACTATAGGGAGAACAATACGACTACGGGAGGGTTTTCTTATGATGACTATAAGACCTACTAAAAGTACAGACTTTGAgg                                                                  <  1:419617/301‑1 (MQ=255)`  
                                                                                                                                                                                                                                                 `agtAAAACTCTGAGGGCTGGGAGACTGAACTTAGGGAACGGACAATATCGATTCCCTGTGGTTGGTAACGCCAAGTTCAACACTGTATACATCTTGTCAGATGAGACTACCCCTCTGAACATCATTGGGTGTGGCTGGGAAGGTAACTACTTACGGAGAAGTTCCGGTATTTAATTAAATATTCTCCCTGTGGTGGCTCGAAATTAATACGACTCACTATAGGGAGAACAATACGACTACGGGAGGGTTTTCTTATGATGACTATAAGACCTACTAAAAGTACAGACTTTGAGGTATTCAc                                                           <  1:70279/299‑1 (MQ=255)`  
                                                                                                                                                                                                                                                       `acTCTGAGGGCTGGGAGACTGAACTTAGGGACCGGACAATATCGATTCCCTGCGGTTGGTAACGCCAAGTTCAACACTGTATACATCTTGTCAGATGAGACTACCCCTCTGAACATCATTGGGTGTGGCTGGGAAGGTAACTACTTACGGAGAAGTTCCGGTATTTAATTAAATATTCTCCCTGTGGTGGCTCGAAATTAATACGACTCACTATAGGGAGAACAATACGACTACGGGAGGGTTTTCTTATGATGACTATAAGACCTACTAAAAGTACAGACTTTGAGGTATTCACTCCGGc                                                     <  1:181778/301‑1 (MQ=255)`  
                                                                                                                                                                                                                                                        `ctctGAGGGCTGGGAGACTGAACTTAGGGACCGGACAATATCGATTCCCTGTGGTTGGTAACGCCAAGTTCAACACTGTATACATCTTGTCAGATGAGACTTCCTCCCTGAACAACATTGGGTGGGGCTGGGAAGGTAACTACTTACGGAGAATTTCCGGTATTTAATTAACTATTCTCCCTGTGGTGGCTCGAAATTAATACGACTCACTTTAGGGTGAACAATAGTACTACGGGAGGGTTTTCTTATGTTGACTCTATGACCTACTACATGACCAGACTTTGTGGTATTCTCTCCGGCt                                                    >  1:79098/1‑301 (MQ=255)`  
                                                                                                                                                                                                                                                            `gatggCTGGGAGACTGAACTTAGGGACCGGACAATATCGATTCCCTGTGGTTGGTAACGCCAAGTTCAACACTGTATACATCTTGTCAGATGAGACTACCCCTCTGAACATCATTGGGTGTGGCTGGGAAGGTAACTACGTACGGAGAAGTTCCGGTATTTAATTAAATGTTCTCCCTGTGGTGGCTCGAAATTAATACGACTCACTATAGGGAGAACAATACGACTACGGGAGGGTTTTCTTATGATGACTATAAGACCTACTAAAAGTACAGACTTTGAGGTATTCACTACGGCTCAcc                                                <  1:506973/298‑1 (MQ=255)`  
                                                                                                                                                                                                                                                              `gggCTGGGAGACTGAACTTAGGGACCGGACAATATCGATTCCCTGCGGTTGGTAACGCCAAGTTCAACACTGTATACATCTTGTCAGATGAGACTACCCCTCTGAACATCATTGGGTGTGGCTGGGAAGGTAACTACTTACGGAGAAGTTCAGGTATTTAATTAAATATTCTCCCTGTGGTGGCTCGAAATTAATACGACTCACTATAGGGAGAACAATACGACTACGGGAGGGTTTTCTTATGATGACTATAAGACCTACTAAAAGTACAGACTTTGAGGTATTCACTccctgtctctta                                              >  1:405290/1‑291 (MQ=255)`  
                                                                                                                                                                                                                                                                   `gggAGACTGAACATAGGGACCGGAAAATATCGATGCCCTGCGGTTGGTAAAGCCAAGGTCAACACTGTATACATCTTGTCAGATGAGACTACCCCTCTGAACATCATTGGGTGTGGCTGGGAAGGTAACTACTTACGGAGAAGTTCCGGTATTTAATTAAATATTCTCCCTGTGGTGGCTCGAAATGAATACGACTCACTATAGGGAGAACAATACGACTACGGGAGGGTTTTCTTATGATGACTATAAGACCTACTAAAAGTACAGACTTTGAGGTATTCACTCCGGCTCACCATGACAt                                         <  1:320377/301‑1 (MQ=255)`  
                                                                                                                                                                                                                                                                     `gagaCTGAACTTAGGGACCGGACAATATCGATTCCCTGCGGTTGGTAACGCCAAGTTCAACACTGTATACATCTTGTCAGATGAGACTACCCCTCTGAACATCATTGGGTGTGGCTGGGAAGGTAACTACTTACGGAGAAGTTCCGGTATTTAATTAAATATTCTCCCTGTGGTGGCTCGAAATTAATACGACTCACTATAGGGAGAACAATACGACTACGGGAGGGTTTTCTTATGATGACTATAAGACCTACTAAAAGTACAGACTTTGAGGTATTCACTCCGGCTCACCATGACATTc                                       >  1:427531/1‑301 (MQ=255)`  
                                                                                                                                                                                                                                                                        `aCTGAACTTAGGGACCGGACAATATCGATTCCCTGTGGTTGGTAACGCCAAGTTCAACACTGTATACATCTTGTCAGATGAGACTACCCCTCTGAACATCATTGGGTGTGGCTGGGAAGGTAACTACTTACGGAGAAGTTCCGGTATTTAATTAAATATTCTCCCTGTGGTGGCTCGAGATTAATACGACTCACTATAGGGAGAACAATACGACTACTGGAGGGATTTCTTATGATGAGTGTAAGACCTACTAAAAGTACAGACTTTGAGGTATTCACTCCGGCTCACCATGCCcttcttt                                    >  1:392621/1‑300 (MQ=255)`  
                                                                                                                                                                                                                                                                             `gcTTAGGGACCGGACAATATCGATTCCCTGCGGTTGGTAACGCCAAGTTCAACACTGTATACTTCTTGTCAGATGAGACTACCCCTCTGAACATCATTGGGTGTGGCTGGGAAGGTAACTACTTACGGAGAAGTTCCGGTATTTAATTAAATATTCTCCCTGTGGTGGCTCGAAATTAATACGACTCACTATAGGGAGAACAATACGACTACGGGAGGGTTTTCTTATGATGACTATAAGACCTACTAAAAGTACAGACTTTGAGGTATTCACTCCGGCTCACCATGACATTCTTGAAGCt                               <  1:270751/300‑1 (MQ=255)`  
                                                                                                                                                                                                                                                                                 `aGGGACCGGACAATATCGATTCCCTGTGGTTGGTAACGCCAAGTTCAACACTGTATACATCTTGTCAGATGAGACTACCCCTCTGAACATCATTGGGTGTGGCTGGGAAGGTAACTACTTACGGAGAAGTTCCGGTATTTAATTAAATATTCTCCCTGTGGTGGCTCGAAATTAATACGACTCACTATAGGGAGAACAATACGACTACGGGAGGGTTTTCTTATGATGACTATAAGACCTACTAAAAGTACAGACTTTGAGGTA‑TCACTCCGGCTCACCATGACATTCTTGAAGCTAAGgc                          <  1:308510/301‑1 (MQ=255)`  
                                                                                                                                                                                                                                                                                        `ggACAATATCGATTCCCTGTGGTTGGTAACGCCAAGTTCAACACTGTATACATCTTGTCAGATGAGACTACCCCTCTGAACATCATTGGGTGTGGCTGGGAAGGTAACTACTTACGGAGAAGTTCCGGTATTTAATTAAATATTCTCCATGTGGTGGCTCGAAATTAATACGACTCACTATAGGGAGAACAATACGACTACGGGAGGGTTTTCTTATGATGACTATAAGACCTACTAAAAGTACAGACTTTGAGGAATTCACTCCGTCTCACCATGACATACTTGAAGCTAAGactccggg                    >  1:51833/1‑296 (MQ=255)`  
                                                                                                                                                                                                                                                                                         `gACAATATCGATTCCCTGTGGTTGGTAACGCAAAGTTCAACACTGTATACATCTTGTCAGATGAGACTACCCCTCTGAACATCATTGGGTGTGGCTGGGAAGGTAACTACTTACGGAGAAGTTCCGGTATTTAATTAAATATTCTCCCTGTGGTGGCTCGAAATTAATACGACTCACTATAGGGAGAACAATACGACTACGGGAGGGTTTTCTTATGATGACTATAAGACATACTAAAAGTACAGCCTTTGAGGTATTCACTCCGGCTCACCATGACATTCTTGAAGCTAAGgcctgtctc                   >  1:297335/1‑294 (MQ=255)`  
                                                                                                                                                                                                                                                                                         `aaCAGTATCGATTCCCTGCGGTTGGTAACGCCAAGTTCAACACTGTATACATCTTGTCAGATGAGACTACCCCTCTGAACATCATTGGGTGTGGCTGGGAAGGTAACTACTTACGGAGAAGTTCCGGTATTTAATTAAATATTCTCCCTGTGGTGGCTCGAAACTAATACGACTCACTATAGGGAGAACAATACGACTACGGGAGGGTTTTCTTATGATGACTATAAGACCTACTAAAAGTACAGACTTTGAGGTATTCACTCCGGCTCACCATGACATTATTGAAGCTAAGGCTGCTGGt                   <  1:233203/300‑1 (MQ=255)`  
                                                                                                                                                                                                                                                                                             `atatTGATTCCCTGTGGTTGGTAACGCCAAGTTCAACACTGTATACATCTTGTCAGATGAGACTACCCCTCTGAACATCATTGGGTGTGGCTGGGAAGGTAACTACTTACGGAGAAGTTCCGGTATTTAATTAAATATTCTCCCTGTGGTGGCACGAAATTAATACGACTCACTATAGGGAGAACAATACGACTACGGGAGGGTTTTCTTATGATGACTATAAGACCTACTAAAAGTACAGACTTTGAGGTATTCACTCCGGCTAACCATGACATTCTTGAAGCTAAGACTGCTGGGATTg               >  1:247610/1‑301 (MQ=255)`  

                                                                                                                                                                                                                                                                                                           `|`                                                                                                                                                                                                                                                                                                             
`ACTCAGCGGTAACTTGGAGGGACGCATGGTGTACATTGGGTTCAACATTAACTTCGTATATGAGTTCTCTAAGTTCCTCATCAAGCAGACTGCCGACGACGGGTCTACCTCCACGGAAGACATTGGGCGCTTACAGTTACGCCGAGCGTGGGTTAACTACGAGAACTCTGGTACGTTTGACATTTATGTTGAGAACCAATCGTCTAACTGGAAGTACACAATGGCTGGTGCCCGATTAGGCTCTAACACTCTGAGGGCTGGGAGACTGAACTTAGGGACCGGACAATATCGATTCCCTGTGGTTGGTAACGCCAAGTTCAACACTGTATACATCTTGTCAGATGAGACTACCCCTCTGAACATCATTGGGTGTGGCTGGGAAGGTAACTACTTACGGAGAAGTTCCGGTATTTAATTAAATATTCTCCCTGTGGTGGCTCGAAATTAATACGACTCACTATAGGGAGAACAATACGACTACGGGAGGGTTTTCTTATGATGACTATAAGACCTACTAAAAGTACAGACTTTGAGGTATTCACTCCGGCTCACCATGACATTCTTGAAGCTAAGGCTGCTGGTATTGAGCCGAGTTTCCC  >  NC_001604.1/26812‑27410`

  

Alignment Legend

Aligned base mismatch/match (shaded by quality score): `ATCG/ATCG` `< 3 ≤` `ATCG/ATCG` `< 9 ≤` `ATCG/ATCG` `< 18 ≤` `ATCG/ATCG` `< 33 ≤` `ATCG/ATCG` `< 38 ≤` `ATCG/ATCG`

Unaligned base: `atcg`    Masked matching base: `atcg`    Alignment gap: `‑`    Deleted base: `‑`
